# Moral distress in pediatric nurses: A scoping review protocol

**DOI:** 10.1371/journal.pone.0312808

**Published:** 2024-10-31

**Authors:** Haiyan Zhou, Huiling Liao, Yuanyuan Huang, Qin Lin, Xin Wang, Huimin Li, Fang Wu, Sha Yang

**Affiliations:** 1 Chengdu Women’s and Children’s Central Hospital, School of Medicine, University of Electronic Science and Technology of China, Chengdu, China; 2 Shulan (Hangzhou) Hospital Affiliated to Zhejiang Shuren University Shulan International Medical College, Hangzhou, China; University of the Witwatersrand, SOUTH AFRICA

## Abstract

**Introduction:**

Recently, moral distress in pediatric nursing has gained academic attention, yet comprehensive literature reviews on this group are scarce.

**Aims:**

This study aims to offer a detailed overview of moral distress among pediatric nurses, focusing on understanding its characteristics, prevalence, underlying causes, and consequences on the quality of patient care.

**Methods:**

Employing a scoping review approach as recommended by the Joanna Briggs Institute, this study will systematically search through PubMed, Scopus, Web of Science, APA PsycInfo, and CINAHL databases using specific search strategies. Titles, abstracts, and full texts will be independently screened by two reviewers according to the eligibility criteria. Relevant data will be extracted, categorized, and subjected to narrative synthesis to draw comprehensive insights.

**Conclusion:**

The anticipated findings of this study will shed light on the nature, frequency, and drivers of moral distress among pediatric nurses, along with its broader implications for healthcare practitioners, organizational practices, and patient care outcomes.

## Introduction

Moral distress, a term first introduced by Andrew Jameton in 1984, refers to the ethical suffering individuals experience when they identify the right course of action but are unable to pursue it [1]. This issue is especially common among nurses, particularly in pediatric settings [2–4]. A survey by Georgina et al. [5] across Midwestern U.S. hospitals found 21% of nurses experienced moral distress. In Canadian pediatric and neonatal ICUs, 58% of healthcare workers faced moral distress, with a higher frequency reported among nurses than physicians [6]. In Australia, Ingrid et al. found that 68% of pediatric hospital nurses faced moral distress, highlighting the challenge of moral distress in pediatric healthcare [7].

Nurses’ central role in preserving patient dignity and integrity often places them at the heart of moral distress [8, 9]. Extensive research highlights the ethical challenges nurses face, such as informed consent, limited medical resource allocation, colleague misconduct, and policies conflicting with patient interests [3, 10]. Pediatric nurses face unique challenges, as they deal with patients of various ages who may find it hard to communicate their needs, especially in end-of-life situations [5]. Unlike adults, children—particularly those with communication barriers related to age or cognitive issues—often find it challenging to express their end-of-life preferences [11]. This situation poses challenges for healthcare providers, who frequently must inform and support the family [7]. Moral distress in pediatric nurses often arises from conflicts among professional ethics, communication difficulties with young patients, high parental involvement, difficult care practices, and rigid administrative protocols [12, 13]. Prioritizing the best interests of young patients amplifies these challenges, necessitating a delicate balance between clinical judgment and ethical principles to achieve optimal care [14–16].

This distress impacts pediatric nurses’ physical and mental well-being, care quality, and professional development. Research indicates that nurses enduring moral distress frequently experience negative emotions, including anger, guilt, sadness, self-doubt, and lowered self-esteem, which profoundly affect their professional interactions [17]. As a result, this distress can lower work engagement and satisfaction, potentially leading to a higher turnover rate [18].

Moral distress among nurses has been a prominent concern globally for over three decades [19]. Recently, the specific moral distress that pediatric nurses encounter is now gaining more academic attention. This expanding area of study encompasses the development and refinement of assessment tools, the analysis of factors that contribute to moral distress, and the investigation of interventions to alleviate these challenges [20]. In the United States, Jessica et al. examined the impact of moral distress on the psychological resilience of pediatric emergency nurses using two distinct scales before the COVID-19 pandemic [21]. Similarly, Soojeong et al. explored the experiences of nurses facing end-of-life care dilemmas in Seoul, South Korea’s neonatal intensive care units (NICUs) [19]. In Canada, Sadie et al. employed qualitative methods to understand how pediatric ICU nurses cope with moral distress, aiming to uncover effective interventions [22]. Moreover, Ventovaara et al. reported from a Finnish pediatric oncology center that an escalation in moral distress among nurses corresponded with a decline in the ethical climate, highlighting the pervasive effects of moral distress across different pediatric care settings [23].

However, comprehensive reviews on moral distress in pediatric nurses are scarce. Although prior research has explored nurses’ moral distress from diverse perspectives—like regional differences [24, 25], adult care settings [24, 26], and the impact of the COVID-19 pandemic [21, 24]—studies specifically targeting pediatric nursing are significantly less common. Annamaria Bagnasco’s team performed a rapid evidence review on pediatric nursing ethics, emphasizing common ethical issues and skills. However, the review’s scope was restricted by the limited number of publications, and it is now viewed as outdated due to the lack of inclusion of more recent advancements in the field [12]. In 2022, a Chinese researcher provided a succinct overview of recent studies on the ethical challenges in pediatric nursing and their influencing factors, yet this review too did not offer a comprehensive analysis [27]. This indicates an urgent need for a thorough review of ethical distress within pediatric care, one that thoroughly examines its key characteristics, prevalence, underlying causes, and impacts of moral distress among this group.

This study seeks to bridge this gap through a scoping review approach, aiming to methodically gather, evaluate, and synthesize available literature on moral distress among pediatric nurses. This study aims to clarify the nature, frequency, and drivers of moral distress among pediatric nurses, Furthermore, it will strive to identify effective strategies for reducing moral distress among pediatric nurses, thereby contributing to the enhancement of both patient care and the nursing profession within pediatric settings.

## Review objectives

To explore the nature and characteristics of moral distress among pediatric nurses.

To assess the degree of moral distress experienced by pediatric nurses.

To identify the factors contributing to moral distress in pediatric nurses.

To examine the outcomes of moral distress on pediatric nurses, their practice, and patient care.

To evaluate and recommend strategies for managing and reducing moral distress among pediatric nurses.

## Methods

### Design

This scoping review will be conducted in accordance with the Joanna Briggs Institute (JBI) guidelines for scoping reviews, involving seven methodical stages: (1) defining the research question, (2) developing the protocol, (3) applying the PCC (Population/Concept/Context) framework, (4) conducting systematic searches, (5) screening studies, (6) extracting and charting relevant data, and (7) synthesizing and reporting the evidence [28]. This protocol will be reported following the Preferred Reporting Items for Systematic Reviews and Meta-Analysis Protocols (PRISMA-P) (S1 Appendix) [29] and the PRISMA Extension for Scoping Reviews (ScR) guidelines (S2 Appendix) [30]. The protocol is registered with Open Science Framework (https://doi.org/10.17605/0SF.10/MBX98). To facilitate the review process, Covidence software and Microsoft Excel will be used for screening and data extraction.

### Review questions

1. What is the nature of moral distress among pediatric nurses?
2. What is the degree of moral distress experienced by pediatric nurses?
3. What factors are related to moral distress in pediatric nurses?
4. What are the outcomes of moral distress in pediatric nurses?
5. What strategies have been suggested to mitigate moral distress?

### Search strategy

The reviewers will adopt the three-step search strategy [28, 29]. The first phase involves searching PubMed to assess and expand the keywords for nurses, moral distress, and pediatrics by reviewing titles and abstracts. The detailed PubMed search strategy is outlined in Table 1. Subsequently, the strategy will be expanded to encompass all index terms and keywords derived from the titles and abstracts of the articles retrieved. The approach will be tailored and adjusted for every information source, including PubMed, Web of Science, Scopus, CINAHL, and APA PsycInfo. In the third step, the reference lists of all studies included in the review will be searched.

**Table 1:** Search strategy in Pubmed.

*DATABASE*	*MEDLINE*
*PLATFORM*	*Pubmed*
*SEARCH*	*query*
*#1*	*"moral distress"[Title/Abstract] OR "moral dilemma* * *"[Title/Abstract] OR "ethical conflict"[Title/Abstract] OR "ethical dilemma* * *"[Title/Abstract] OR "moral disorder" [Title/Abstract] OR "ethical issue* * "[Title/Abstract] OR "ethical distress"[Title/Abstract] OR "moral conflict"[Title/Abstract] OR "moral issue*"[Title/Abstract]
*#2*	(nurs*[Title/Abstract]) AND ((paed*[Title/Abstract] OR pediat*[Title/Abstract] OR child* Neonat*[Title/Abstract] OR newborns[Title/Abstract] OR infant[Title/Abstract]) OR ("Pediatrics"[Mesh]))
*#3*	*#1 AND #2*

* indicates a wildcard to identify terms that start with the specified string.

#### Eligibility criteria

Following JBI guidelines, we established inclusion criteria using the PCC approach, considering participants, concept, context, and evidence types.

Inclusion criteria

Population: This review will include nurses with diverse demographic characteristics such as age (20–55 years old), gender, work experience(≥1 year), educational level, and geographical location.

Concept: The core concept of this review is moral distress, with a focus on its prevalence, assessment methods, risk factors, consequences, and interventions to mitigate it.

Context: The scope of this review will be confined to research carried out within pediatric department environments.

Evidence source types: This review will include peer-reviewed, full-text research papers from various countries, covering qualitative, quantitative, and mixed-methods approaches.

Exclusion criteria include duplicated publications, articles with incomplete data, literature reviews, case reports, experimental protocols, opinions, and articles in languages other than English.

### Data selection

**Fig 1:**
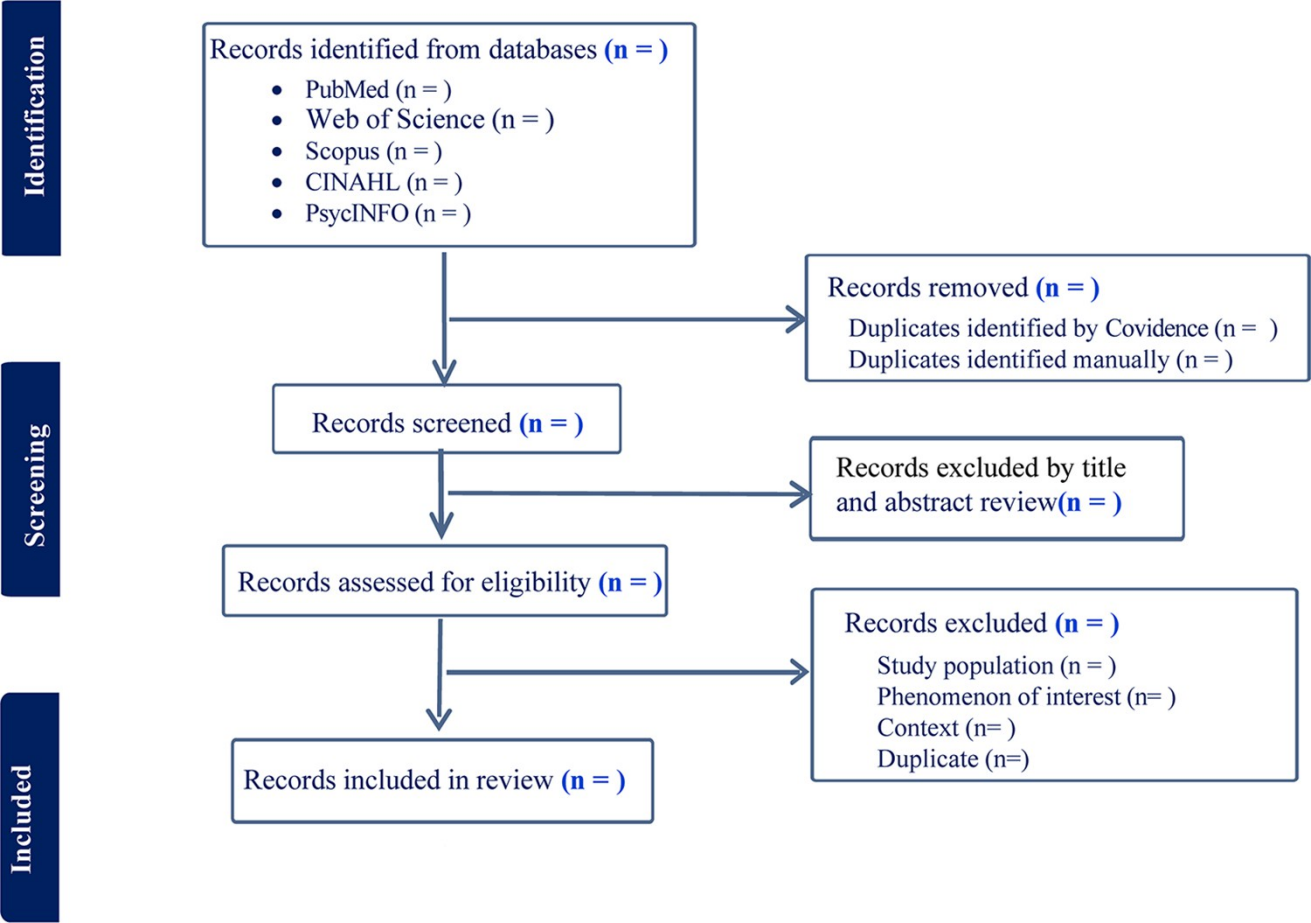
Flow chart of study selection.

After retrieval, all identified articles will be gathered and uploaded to Covidence, a platform tailored for optimizing the screening and extraction of data in systematic reviews. Titles and abstracts will be independently evaluated by two researchers to eliminate studies not aligning with the inclusion criteria, succeeded by an in-depth review of the full texts for further refinement. Every phase of the review process will be independently carried out and cross-checked by the two researchers, with any discrepancies resolved through discussion or consultation with a third party, if needed. This methodology will be systematically recorded following the PRISMA flow diagram (Fig 1).

### Data extraction

Data extraction from selected articles will be performed by two independent reviewers using Microsoft Excel. The initial draft will consist of items related to the author, publication year, journal, study objectives, setting, study design, samples, prevalence and assessment of moral distress, factors, interventions, and outcomes. The spreadsheet will be updated and refined throughout the data selection process to ensure the efficient capture of all relevant information. Should there be alterations to the tool, previously analyzed texts will undergo a thorough reassessment. The final scoping review report will detail any modifications to the tool. Discrepancies between reviewers will be resolved through group discussions or by consulting a third reviewer.

### Data synthesis

Data synthesis will employ a rigorous, systematic approach that integrates both quantitative and qualitative analyses. Quantitative data will be summarized using descriptive statistics—mean, median, standard deviation, and range—to illustrate the prevalence of moral distress across studies. Tables will be used to visually present prevalence rates, assessment methodologies, and associated factors. Qualitative data will undergo content analysis with systematic coding to identify key themes and patterns, exploring the contextual and nuanced dimensions of moral distress, including its determinants and impacts. This integrated approach will yield a comprehensive framework, offering a nuanced understanding of moral distress’s prevalence, determinants, and consequences by combining statistical trends with qualitative insights.

## Ethics consideration

This study does not require formal ethical approval as the scoping review will only use published data. The findings of this study will be disseminated through academic papers and conference presentations.

## Limitations

This review has several limitations. Firstly, it will not assess the methodological rigor of the included studies. The review is restricted to papers published in English, which means that relevant articles published in other languages will be excluded. Additionally, only peer-reviewed, full-text research papers will be included, while gray literature and articles lacking sufficient detail will be omitted.

## Discussion and conclusion

Pediatric nurses inherently face complex ethical challenges due to its specialized focus and unique characteristics. This scoping review protocol delineates the methodology to systematically investigate the prevalence, factors, and consequences of ethical dilemmas in pediatric nurses and to identify effective interventions. To our knowledge, this is the first systematic scoping review focused on moral distress within this specific group. Through the analysis of definitions and concepts across diverse studies, this review aims to uncover the scope, trends, and gaps in the literature, facilitating a more profound understanding of moral distress in pediatric nurses. The findings of this review will offer crucial insights for healthcare policymakers and administrators to enhance coping strategies and develop targeted interventions to alleviate moral distress in pediatric nurses.

## Supporting information

S1 Appendix PRISMA-P checklist*. (PDF)

S2 Appendix PRISMA-ScR checklist. (PDF)
